# An artificial intelligence system for comprehensive pathologic outcome prediction in early gastric cancer through endoscopic image analysis (with video)

**DOI:** 10.1007/s10120-024-01524-3

**Published:** 2024-07-02

**Authors:** Seunghan Lee, Jiwoon Jeon, Jinbae Park, Young Hoon Chang, Cheol Min Shin, Mi Jin Oh, Su Hyun Kim, Seungkyung Kang, Su Hee Park, Sang Gyun Kim, Hyuk-Joon Lee, Han-Kwang Yang, Hey Seung Lee, Soo-Jeong Cho

**Affiliations:** 1 Department of Internal Medicine and Liver Research Institute, Seoul National University College of Medicine, 101 Daehak-Ro, Jongno-Gu, Seoul, 03080 Republic of Korea; 2 Ainex Corporation, Seoul, Republic of Korea; 3 Department of Internal Medicine, Seoul National University Bundang Hospital, Seoungnam-Si, Gyeonggi-Do, Republic of Korea; 4 Center for Health Promotion and Optimal Aging, Seoul National University Hospital, Seoul, Republic of Korea; 5 Department of Surgery, Seoul National University College of Medicine, Seoul, Republic of Korea; 6 Department of Pathology, Seoul National University College of Medicine, Seoul, Republic of Korea

**Keywords:** Early gastric cancer, Artificial intelligence, Convolutional neural network, Endoscopy

## Abstract

**Background:**

Accurate prediction of pathologic results for early gastric cancer (EGC) based on endoscopic findings is essential in deciding between endoscopic and surgical resection. This study aimed to develop an artificial intelligence (AI) model to assess comprehensive pathologic characteristics of EGC using white-light endoscopic images and videos.

**Methods:**

To train the model, we retrospectively collected 4,336 images and prospectively included 153 videos from patients with EGC who underwent endoscopic or surgical resection. The performance of the model was tested and compared to that of 16 endoscopists (nine experts and seven novices) using a mutually exclusive set of 260 images and 10 videos. Finally, we conducted external validation using 436 images and 89 videos from another institution.

**Results:**

After training, the model achieved predictive accuracies of 89.7% for undifferentiated histology, 88.0% for submucosal invasion, 87.9% for lymphovascular invasion (LVI), and 92.7% for lymph node metastasis (LNM), using endoscopic videos. The area under the curve values of the model were 0.992 for undifferentiated histology, 0.902 for submucosal invasion, 0.706 for LVI, and 0.680 for LNM in the test. In addition, the model showed significantly higher accuracy than the experts in predicting undifferentiated histology (92.7% vs. 71.6%), submucosal invasion (87.3% vs. 72.6%), and LNM (87.7% vs. 72.3%). The external validation showed accuracies of 75.6% and 71.9% for undifferentiated histology and submucosal invasion, respectively.

**Conclusions:**

AI may assist endoscopists with high predictive performance for differentiation status and invasion depth of EGC. Further research is needed to improve the detection of LVI and LNM.

**Supplementary Information:**

The online version contains supplementary material available at 10.1007/s10120-024-01524-3.

## Introduction

Gastric cancer is the fifth most common malignancy and the fourth leading cause of cancer-related death worldwide [1]. Although radical surgery was traditionally the only curative treatment for gastric cancer, recent advances in endoscopic resection have demonstrated favorable clinical outcomes in early gastric cancer (EGC), concurrently improving quality of life for patients by preserving the stomach [2].

Endoscopic submucosal dissection (ESD) is considered curative for EGC without lymph node metastasis (LNM). Owing to the lack of reliable imaging methods to precisely detect LNM in EGC [3, 4], current guidelines recommend curative criteria for ESD based on pathologic features in resected specimens associated with a minimal risk of LNM [5, 6]. These factors include the differentiation status, invasion depth, and lymphovascular invasion (LVI) of the tumor. Since these characteristics are confirmed postoperatively, the accurate prediction of pathologic outcomes before treatment is essential to select the optimal curative approach between endoscopic and surgical resection.

Endoscopists perform forceps biopsies with assistance of magnifying endoscopy with narrow-band imaging (ME-NBI) to evaluate differentiation status, and endoscopic ultrasonography (EUS) to detect submucosal invasion before deciding the treatment strategy for EGC. However, previous studies have revealed significant histologic discrepancies between biopsies and resected specimens, potentially leading to non-curative ESD or missed opportunities for ESD in surgical cases [7–9]. In addition, EUS is not superior to conventional endoscopy in determining the invasion depth of EGC, with an accuracy of approximately 70% [10–12]. Therefore, a detailed assessment of endoscopic features by physicians is essential for predicting pathologic results in EGC.

With advancements in deep learning methods, recent studies have proposed artificial intelligence (AI) models for detecting and characterizing EGC in endoscopic images, aiming to assist physicians in evaluating endoscopic features [13]. This includes our previous study, where we developed an AI model which can detect EGC in endoscopic videos [14]. Although several models have been developed to assess the invasion depth of EGC using endoscopic images, there remains a need for further research into AI-assisted pathologic prediction for EGC to enhance the performance in video analysis [15, 16]. Moreover, to the best of our knowledge, no previous study has explored the capability of AI in predicting LVI or LNM based on endoscopic images or videos.

Therefore, this study aimed to develop and evaluate an AI model that comprehensively predicts the postoperative pathologic results of EGC, including the differentiation status, invasion depth, LVI, and LNM, based on preoperative white-light endoscopic images and videos.

## Methods

The AI model developed in this study is an extension of the ENAD CAD-G, a convolutional neural network (CNN)-based model for detecting and classifying gastric lesions in endoscopic videos, as demonstrated in our previous study [14].

### Study design and datasets

**Fig. 1:**
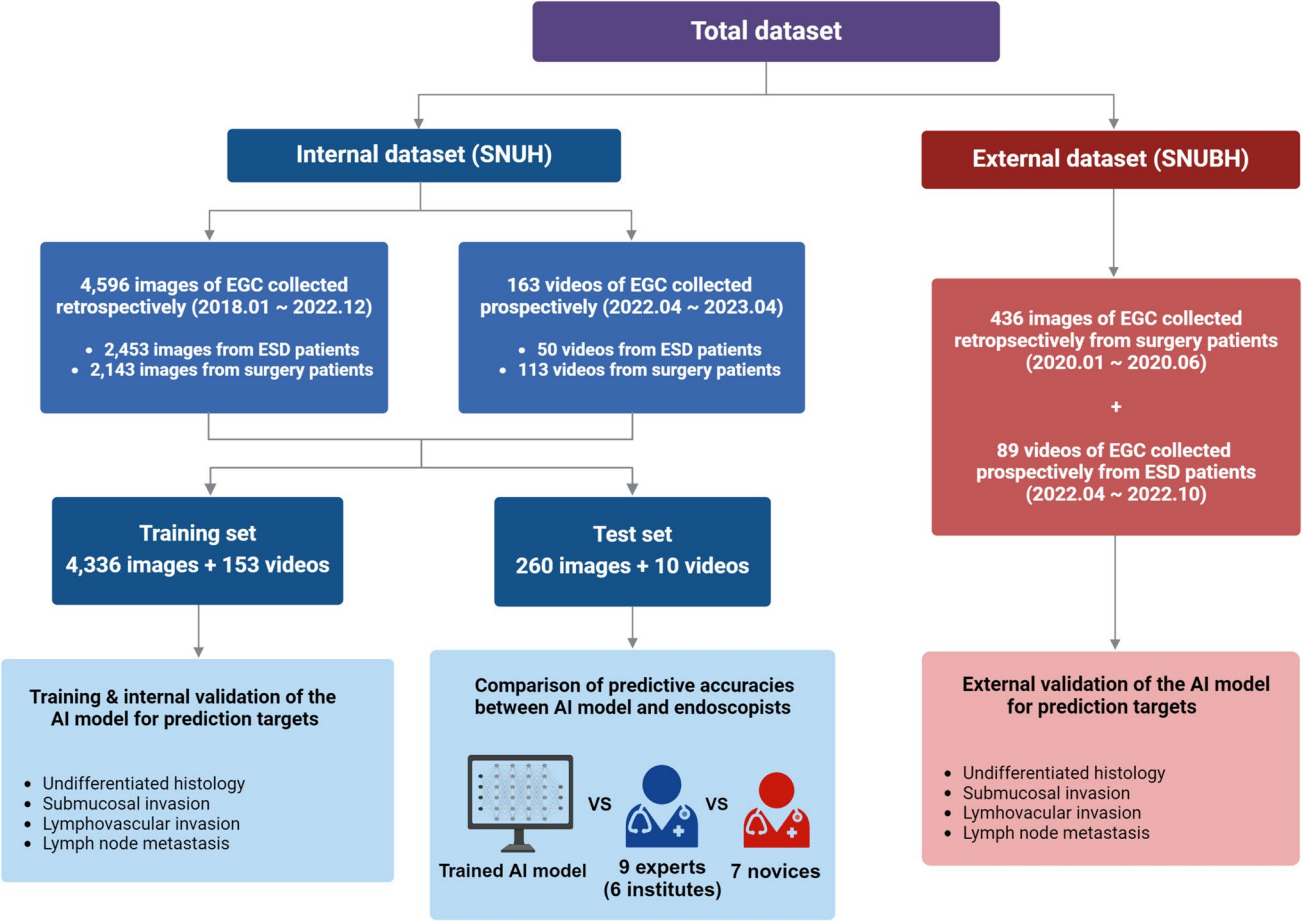
Flow diagram of study design and datasets. *SNUH* Seoul National University Hospital, *SNUBH* Seoul National University Bundang Hospital, *EGC* early gastric cancer, *ESD* endoscopic submucosal dissection, *AI* artificial intelligence

Figure 1 shows an overview of the study design and datasets. The total dataset of endoscopic images and videos was divided into an internal dataset used for training, internal validation, and testing and an external dataset employed for the external validation of the AI model.

For the internal dataset, we retrospectively collected 4,596 preoperative white-light endoscopic images of EGC from patients who underwent ESD or radical surgery between January 2018 and December 2022 at Seoul National University Hospital (SNUH), a tertiary hospital in the Republic of Korea. To assess the performance of the AI model in videos, we prospectively included 163 white-light endoscopic videos of patients referred from community clinics who underwent ESD or surgical resection for EGC between April 2022 and April 2023. For the external dataset, we used 436 images retrospectively collected from patients who underwent surgery for EGC between January 2020 and June 2020, and 89 videos prospectively collected from patients who underwent ESD for EGC between April 2022 and October 2022 at another tertiary hospital, Seoul National University Bundang Hospital (SNUBH), Republic of Korea.

This study was conducted in accordance with the Declaration of Helsinki and was approved by the ethics committees of the participating hospitals (IRB No. 2109–048-1253 at SNUH and IRB No. 2201–735-405 at SNUBH). Written informed consent was obtained from all prospectively enrolled patients who provided endoscopic videos. The requirement for informed consent was waived for the patients whose retrospective images were included in this study.

### Preparation of endoscopic images

Supplementary Figure S1 shows the process of preparing endoscopic images before training the model. We retrospectively investigated the medical records of 1,617 patients who underwent ESD and 1,641 patients who underwent radical surgery for EGC at SNUH between 2018 and 2022. All preoperative white-light endoscopic images of the patients were reviewed by five endoscopists from SNUH, who selected images that best characterized the target lesions (two or three images per lesion) and excluded images with low resolution or blurring. Patients who underwent additional surgery after non-curative ESD were considered as surgical patients. Patients who had received any previous endoscopic treatment for the target lesions before ESD or had undergone ESD at another hospital before surgery were excluded. Patients with inconclusive pathologic results or those who did not undergo preoperative endoscopies at SNUH were also excluded. Finally, 2,453 images from 1,031 (51.4% of total patients) patients who underwent ESD and 2,143 images from 975 (48.6% of total patients) patients who underwent surgery were included in the internal dataset.

### Patient enrollment of endoscopic videos

We prospectively enrolled patients who were diagnosed with gastric dysplasia or EGC on initial biopsies, underwent ESD or radical surgery, and were confirmed with EGC based on the pathological reports of the resected specimens. The indication for ESD was one of the following conditions: i) differentiated-type EGC with tumor size ≤ 2 cm and endoscopically suspected mucosal cancer without ulceration or ii) high-grade dysplasia [17]. We excluded patients who had previously undergone gastrectomy and those with contraindications for biopsy due to bleeding tendency or anticoagulant use. All endoscopic examinations were performed preoperatively using standard video endoscopes (GIF-Q260, GIF-H260, or GIF-H290; Olympus Medical Systems, Tokyo, Japan). Consequently, 163 (50 ESD patients and 113 surgery patients) patients from SNUH and 89 ESD patients from SNUBH were included in the study, and their endoscopic videos were provided. All ESD procedures were performed by experienced endoscopists following a standardized protocol [18]. The surgical procedures were based on standard gastrectomy with D1 + or D2 lymph node dissection [5].

### Pathologic definitions

The pathologic characteristics of EGC in the images and videos were obtained from the pathological reports of specimens resected by ESD or surgery based on the 2022 Korean gastric cancer treatment guidelines [5]. Expert pathologists assessed all resected specimens. Differentiated-type EGC includes papillary, well, or moderately differentiated tubular adenocarcinoma, whereas undifferentiated-type EGC includes poorly differentiated adenocarcinoma, signet ring cell carcinoma, and mucinous carcinoma. In cases of mixed-type gastric cancer, the classification was determined by the histological type of the predominant lesion [19]. Submucosal invasion < 500 µm was defined as SM1 and submucosal invasion ≥ 500 µm was defined as SM2. The status of LNM in the resected specimens was also investigated in the surgical cases.

### Distribution of target lesions in datasets

Table 1 summarizes the pathologic characteristics of EGC in the datasets. The training set comprised 19.6% (850/4,336) of images and 31.3% (48/153) of videos for undifferentiated histology, 23.7% (1,027/4,336) of images and 27.5% (42/153) of videos for submucosal invasion, 7.5% (327/4,332) of images and 6.5% (10/153) of videos for LVI, and 9.5% (187/1,962) of images and 5.7% (6/105) of videos for LNM.

**Table 1:** Pathologic characteristics of early gastric cancer in endoscopic images and videos across datasets

		Internal dataset				External dataset	
		Training set		Test set			
		Images (patients)	Videos	Images (patients)	Videos	Images (patients)	Videos
Total		4336 (1906)	153	260 (100)	10	436 (153)	89
Differentiation status	Differentiated	3486 (1499)	105	147 (50)	5	205 (72)	88
	Undifferentiated	850 (407)	48	113 (50)	5	231 (81)	1
Invasion depth	Mucosal	3309 (1419)	111	114 (50)	6	248 (87)	78
	Submucosal	1027 (487)	42	136 (50)	4	188 (66)	11
Lymphovascular invasion	Positive	327 (154)	10	50 (19)	1	50 (18)	4
	Negative	4005 (1750)	143	210 (81)	9	386 (135)	85
Lymph node metastasis	Positive	187 (83)	6	32 (9)	3	57 (20)	N/A
	Negative	1755 (819)	99	169 (64)	5	379 (133)	N/A

The test set comprised 43.5% (113/260) of images and 50% (5/10) of videos for undifferentiated histology, 52.3% (136/260) of images and 40% (4/10) of videos for submucosal invasion, 19.2% (50/260) of images and 10% (1/10) of videos for LVI, and 15.9% (32/201) of images and 37.5% (3/8) of videos for LNM. In addition, the external dataset included 53.0% (231/436) of images and 1.1% (1/89) of videos for undifferentiated histology, 43.1% (188/436) of images and 12.4% (11/89) of videos for submucosal invasion, 11.5% (50/436) of images and 4.5% (4/89) of videos for LVI, and 13.1% (57/436) of images for LNM.

### Training and internal validation of the AI model

The training set comprised 4,336 images and 153 videos, which were used to train and internally validate the AI model (Fig. 1). For the internal validation, the images and videos in the dataset were randomly divided into five subsets. Four subsets were used for training, and the remaining subset, was used for validation to calculate the predictive performance of the trained model. This cross-validation process was conducted five times to ensure comprehensive evaluation of all images and videos in the set.

**Fig. 2:**
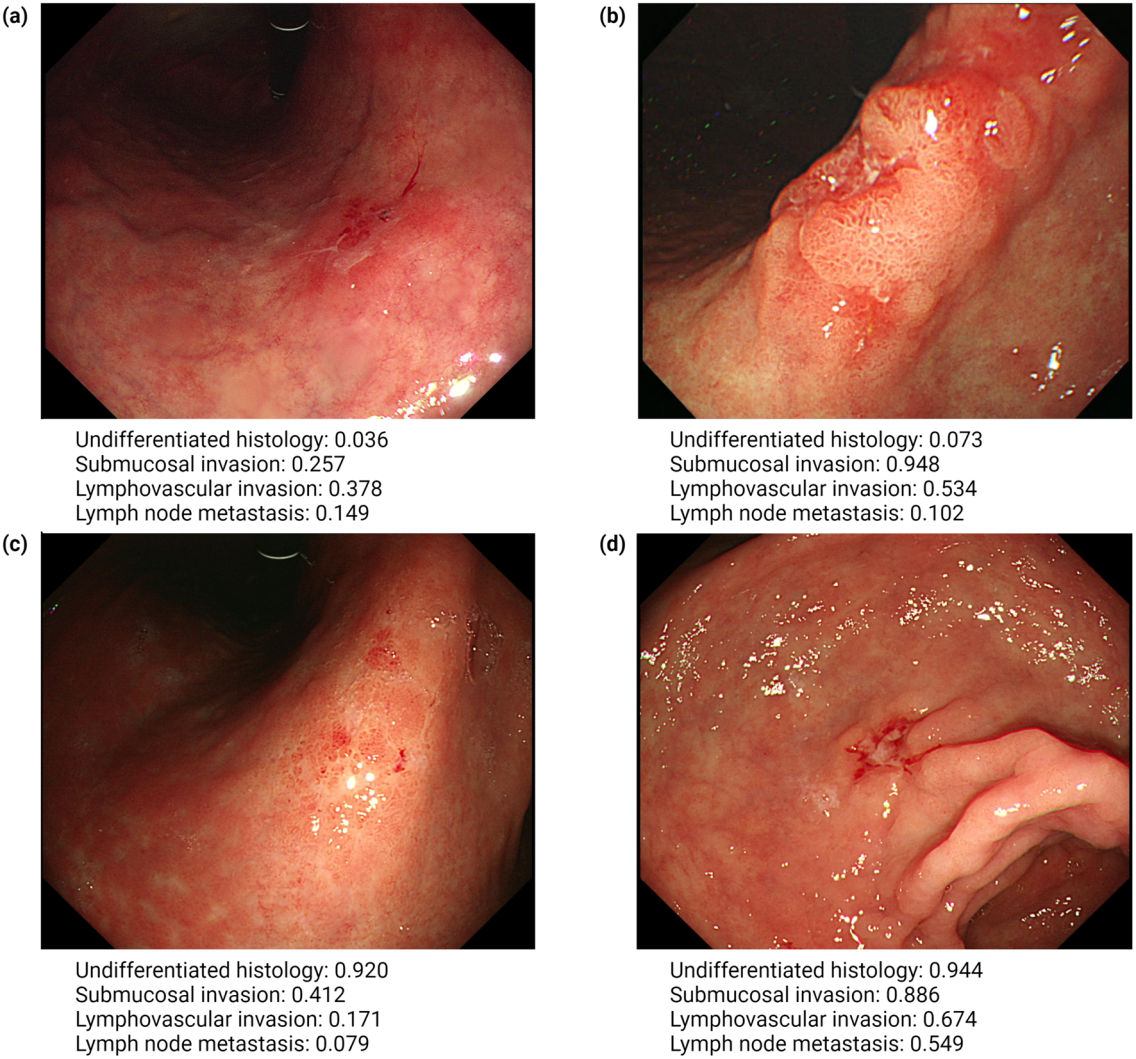
Representative examples of pathologic predictions by the AI model in endoscopic images. Each endoscopic image contains one lesion of EGC with the following pathologic characteristics. **a** Differentiated-type EGC of mucosal invasion without both LVI and LNM. **b** Differentiated-type EGC of submucosal invasion with positive LVI and negative LNM. **c** Undifferentiated-type EGC of mucosal invasion without both LVI and LNM. **d** Undifferentiated-type EGC of submucosal invasion with both LVI and LNM. The provided confidence levels represent the probabilistic evaluation of the AI model for predicting postoperative pathologic results of EGC in the images. *AI* artificial intelligence, *EGC* early gastric cancer, *LVI* lymphovascular invasion, *LNM* lymph node metastasis

The AI model was based on CNN architecture and utilized Efficientnetb0 to evaluate the pathologic characteristics of target lesions in endoscopic images [20]. The model employed a soft voting method to categorize these lesions into distinct predictive classes: differentiation status (differentiated or undifferentiated), invasion depth (mucosal or submucosal), LVI (positive or negative), and LNM (positive or negative). A generative model using Stylegan2 was integrated to enhance predictive performance of the model and increase its sensitivity [21]. Representative images analyzed by the model are presented in Fig. 2.

**Fig. 3:**
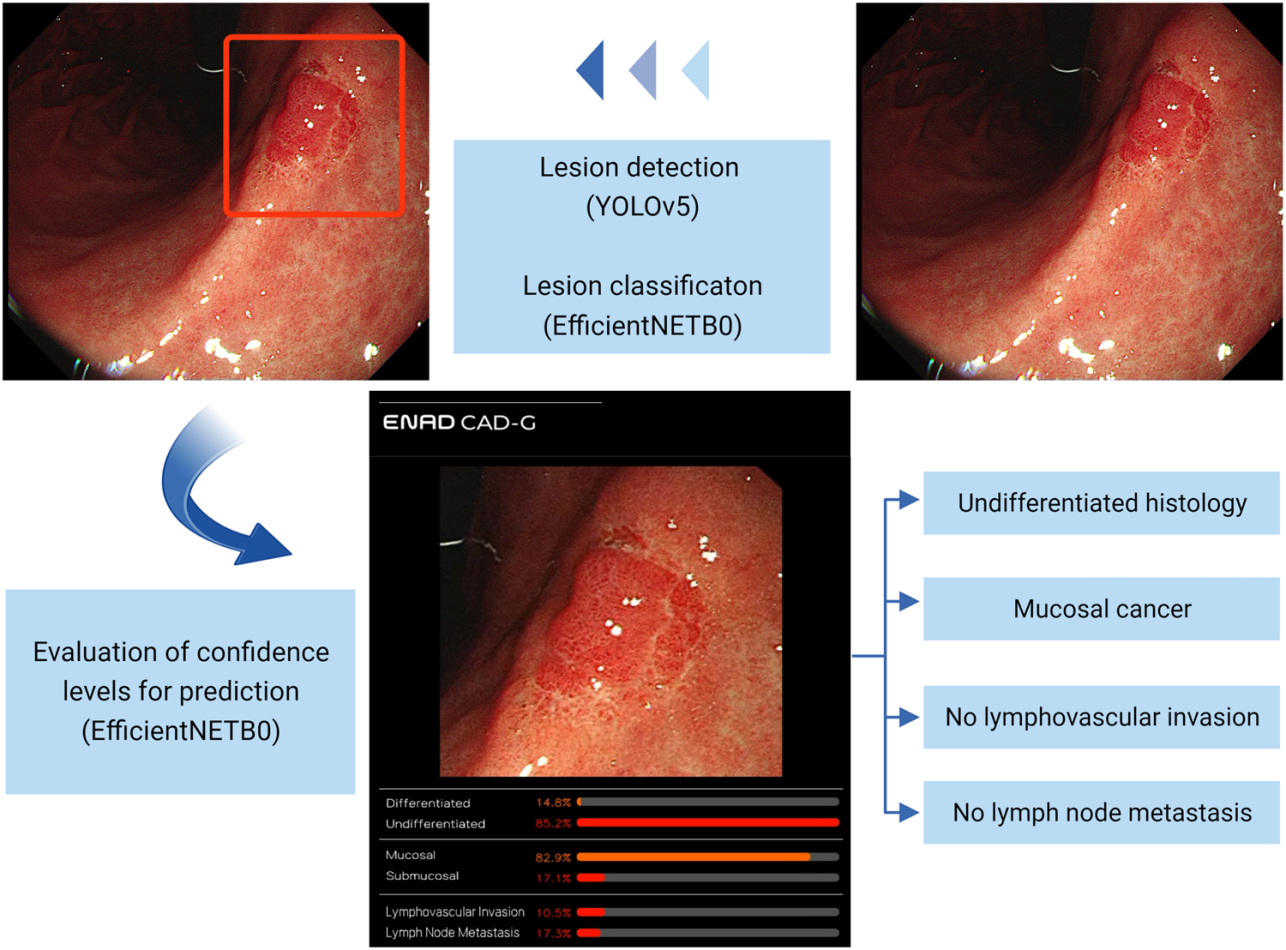
Schematic diagram for AI-based pathologic prediction in endoscopic videos. Initially, the gastric lesion is identified and outlined with red boundaries (cropped) with the lesion detection model. Subsequently, the cropped lesion is categorized as either cancer, adenoma, or non-neoplastic lesion by the lesion classification model. For lesions classified as cancer, the model computes confidence levels to predict differentiation status, invasion depth, lymphovascular invasion, and lymph node metastasis. Finally, the lesion is categorized into distinct pathologic classes utilizing a soft voting method. *AI* artificial intelligence, *EGC* early gastric cancer

Figure 3 shows a schematic diagram of the evaluation of the endoscopic videos. Initially, gastric lesions within the videos were recognized and outlined with boundaries (cropped), using a lesion detection model based on YOLOv5 developed in our previous study [14]. Subsequently, the cropped images were categorized as cancer, adenoma, or non-neoplastic lesions using a lesion classification model that employs EfficientNETB0 [14]. The AI model then calculated the confidence levels for pathologic predictions of the identified cancers. Finally, the model utilized a soft voting method to determine the pathologic classifications of the cancers. Therefore, the cut-off value for considering AI prediction as correct was set at 50%, and this method was utilized in all the validation process.

### Testing the performance of the AI model and endoscopists

A test set was designed to evaluate and compare the predictive performances of the AI model and endoscopists, using 260 endoscopic images and 10 videos distinct from the training set. Sixteen endoscopists, comprising nine experts and seven novices, participated in the test and predicted the differentiation status, invasion depth, LVI, and LNM of the target lesions in the test set. The test was performed as a questionnaire comprising 100 questions, each including two or three images of one target lesion, and additional 10 questions with video clips, each containing one target lesion, as shown in Supplementary Figure S2. Each novice endoscopist had 1 year of experience in upper endoscopy prior to this study, conducted at least 500 procedures independently, and diagnosed EGC in clinical practice. The experts comprised endoscopists from six tertiary hospitals in the Republic of Korea who had over 10 years of experience performing gastric ESD before this study and held positions of associate professor or higher.

### Statistical analyses

The gold standard for prediction was derived from postoperative pathological reports of specimens obtained from ESD or surgery. Accuracy, sensitivity, specificity, and positive and negative predictive values of the predictions were calculated. The prediction metrics were presented as means with 95% confidence intervals and were compared using the Mann–Whitney U test. Receiver operating characteristic (ROC) curves and the corresponding area under the ROC curve (AUC) values for the AI model were calculated. The accuracies of the AI model and those of all the experts were compared using the McNemar’s test. The accuracies of the experts and novices were compared using the Mann–Whitney U test. All statistical tests were two-sided, and *P* < 0.05 were considered significant. Statistical analyses were performed using the R software 4.2.3. for Windows.

## Results

### Baseline characteristics of patients

Detailed clinical information of enrolled patients and pathologic characteristics of lesions in the datasets are presented in Supplementary Table S1. Baseline characteristics were comparable across the datasets, including mean age (64.5 years vs. 63.6 years vs. 63.5 years), male sex (68.7% vs. 59.1% vs. 69.0%), and mean tumor size (21.0 mm vs. 24.3 mm vs. 22.7 mm).

### Internal validation of the AI model with images and videos

Table 2 presents the predictive metrics (per image or frame) of the AI model after training with images and videos. Based on images, the model achieved mean predictive accuracies of 91.9% (sensitivity, 85.3%; specificity, 94.1%) for undifferentiated histology, 88.4% (sensitivity, 82.4%; specificity: 90.2%) for submucosal invasion, 84.7% (sensitivity, 24.2%; specificity, 96.2%) for LVI, and 86.8% (sensitivity, 27.4%; specificity, 94.0%) for LNM.

**Table 2:** Internal validation of trained AI model with endoscopic images and videos

Prediction target	Image–based mean, (95% CI)	Video-based mean, (95% CI)	*P* value
Undifferentiated histology			
Accuracy (%)	91.9 (91.0–92.9)	89.7 (86.5–92.9)	0.209
Sensitivity (%)	85.3 (82.6–88.0)	83.4 (72.3–94.5)	0.841
Specificity (%)	94.1 (92.1–96.0)	92.1 (88.7–95.5)	0.222
PPV (%)	81.1 (72.5–89.8)	79.4 (62.4–96.5)	0.690
NPV (%)	95.3 (94.0–96.7)	86.9 (76.8–97.0)	0.151
Submucosal invasion			
Accuracy (%)	88.4 (87.8–89.0)	88.0 (83.8–92.3)	0.834
Sensitivity (%)	82.4 (76.6–88.1)	80.4 (64.7–96.0)	0.996
Specificity (%)	90.2 (87.8–92.6)	91.1 (86.4–95.8)	0.996
PPV (%)	72.6 (67.6–78.1)	71.7 (67.9–85.5)	0.841
NPV (%)	94.3 (92.7–95.8)	93.1 (89.2–97.1)	0.841
Lymphovascular invasion			
Accuracy (%)	84.7 (79.7–89.8)	87.9 (80.7–95.0)	0.310
Sensitivity (%)	24.2 (15.6–32.8)	20.0 (11.5–28.5)	0.203
Specificity (%)	96.2 (94.9–97.5)	97.0 (94.3–99.7)	0.590
PPV (%)	53.6 (44.3–62.9)	44.3 (32.5–56.1)	0.537
NPV (%)	87.0 (81.9–92.1)	90.2 (86.4–94.0)	0.398
Lymph node metastasis			
Accuracy (%)	86.8 (85.3–88.2)	92.7 (87.7—97.7)	0.008
Sensitivity (%)	27.4 (20.1–34.7)	16.7 (4.1–22.6)	0.085
Specificity (%)	94.0 (90.2–97.9)	96.5 (89.3–100)	0.151
PPV (%)	37.3 (20.5–54.2)	27.0 (16.3–37.7)	0.672
NPV (%)	91.4 (88.8–94.1)	95.7 (91.1–100)	0.151

AI, artificial intelligence; PPV, positive prediction value; NPV, negative prediction value; CI, confidence interval

For videos, the model demonstrated mean predictive accuracies of 89.7% (sensitivity, 83.4%; specificity, 92.1%) for undifferentiated histology, 88.0% (sensitivity, 80.4%; specificity, 91.1%) for submucosal invasion, 87.9% (sensitivity, 20.0%; specificity, 97.0%) for LVI, and 92.7% (sensitivity, 16.7%; specificity, 96.5%) for LNM.

There was no significant difference in the performance of the AI model between images and videos, except for the accuracy of predicting LNM, which was significantly higher for the videos (*P* = 0.008).

### Performance of the AI model according to pathologic characteristics of EGC

Supplementary Table S2 shows the performance of the AI model for endoscopic images according to the differentiation status of the target lesion within the training set. For differentiated-type EGC, the model exhibited mean accuracies of 91.1% (sensitivity, 82.3%; specificity, 89.2%) for submucosal invasion, 83.5% (sensitivity, 27.3%; specificity, 95.4%) for LVI, and 90.1% (sensitivity, 29.7%; specificity, 96.3%) for LNM. For undifferentiated-type EGC, the model demonstrated mean accuracies of 87.5% (sensitivity, 79.3%; specificity, 93.3%) for submucosal invasion, 88.1% (sensitivity, 10.4%; specificity, 98.6%) for LVI, and 83.8% (sensitivity, 26.9%; specificity, 92.6%) for LNM. The model presented a significantly higher accuracy in differentiated-type EGC than in undifferentiated-type EGC for predicting submucosal invasion (*P* = 0.008) and LNM (*P* = 0.016).

Supplementary Table S3 shows the performance of the AI model for predicting LVI and LNM based on the invasion depth of EGC in the training set. For mucosal cancer, the model exhibited mean accuracies of 89.6% (sensitivity, 3.0%; specificity, 98.6%) for LVI and 96.1% (sensitivity, 9.4%; specificity, 97.4%) for LNM. For submucosal cancer, the model demonstrated mean accuracies of 63.0% (sensitivity, 35.9%; specificity, 83.3%) for LVI and 72.5% (sensitivity, 38.7%; specificity, 81.4%) for LNM. For submucosal cancer with SM2 invasion, the model presented mean accuracies of 64.7% (sensitivity, 41.4%; specificity, 81.5%) for LVI and 73.3% (sensitivity, 39.9%; specificity, 82.4%) for LNM.

### Comparison of the predictive accuracies between AI model and endoscopists

**Fig. 4:**
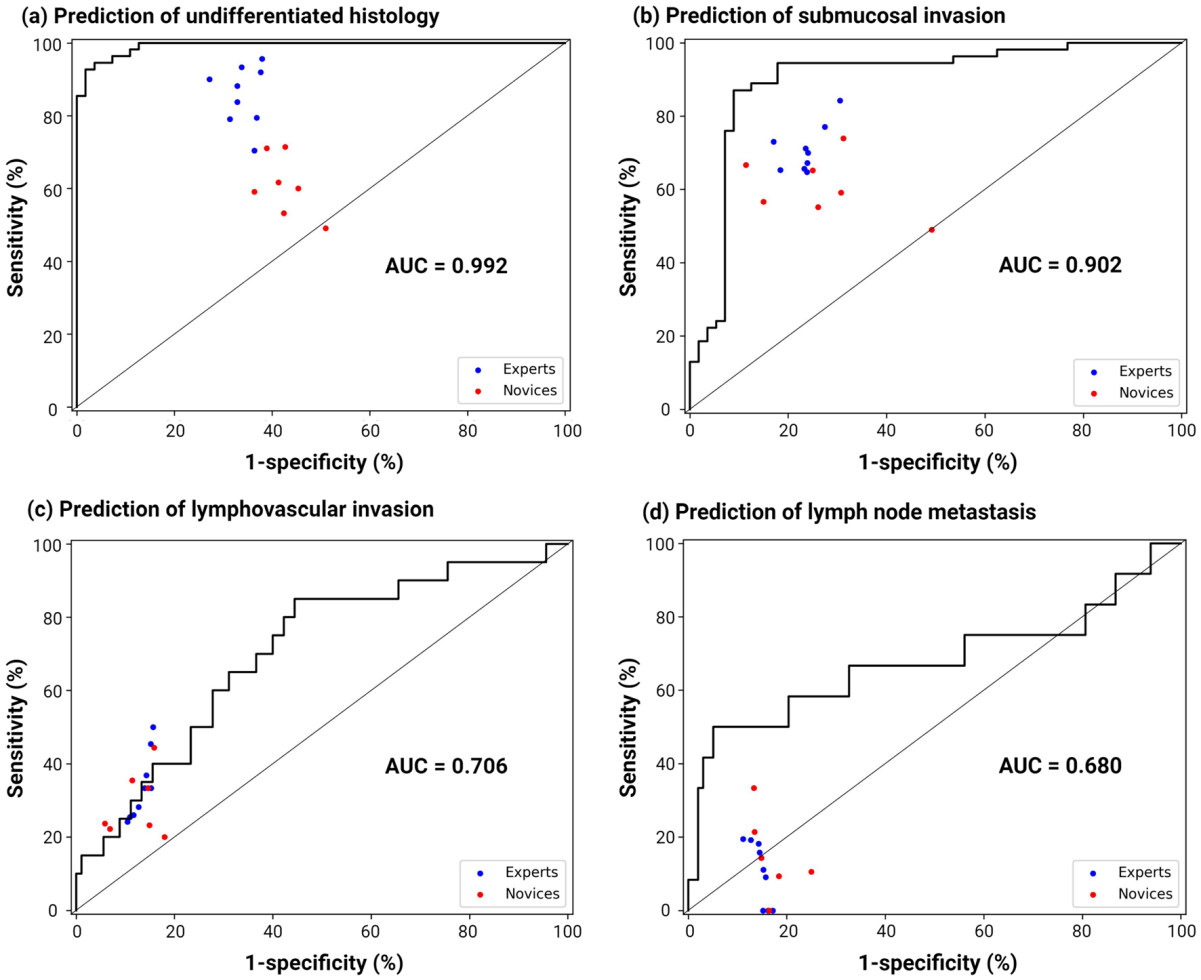
Receiver operating characteristics curves for the AI model in the test set.** a** Prediction of undifferentiated histology (AUC = 0.992). **b** Prediction of submucosal invasion (AUC = 0.902). **c** Prediction of lymphovascular invasion (AUC = 0.706). **d** Prediction of lymph node metastasis (AUC = 0.680). Blue dots (*n* = 9) and red dots (*n* = 7) indicate the performance of the experts and novices, respectively. *AI* artificial intelligence, *AUC* area under the curve

Figure 4 shows the ROC curves demonstrating the performance of the AI model with the performance of the endoscopists, presented as dots (blue = expert, red = novice) in the test set. The AUC values of the model were 0.992 for undifferentiated histology, 0.902 for submucosal invasion, 0.706 for LVI, and 0.680 for LNM. All dots representing the performance of the endoscopists were positioned below the curves for predicting undifferentiated histology, submucosal invasion, and LNM.

Table 3 summarizes the performances of the AI model and endoscopists in the test. The model exhibited accuracies of 92.7% for undifferentiated histology, 87.3% for submucosal invasion, 76.4% for LVI, and 87.7% for LNM. The experts reported mean accuracies of 71.6% for undifferentiated histology, 72.6% for submucosal invasion, 69.7% for LVI, and 72.3% for LNM. The model showed significantly higher accuracy than the experts in predicting undifferentiated histology (*P* ≤ 0.001), submucosal invasion (*P* ≤ 0.012), and LNM (*P* ≤ 0.001). The experts showed significantly higher accuracy than the novices in identifying undifferentiated histology (*P* = 0.001) and submucosal invasion (*P* = 0.019). However, there was no significant difference between the experts and novices in detecting LVI (*P* = 0.525) and LNM (*P* = 0.790).

**Table 3:** Comparison of predictive accuracies between AI model and endoscopists in the test set

Prediction target	AI model (n/N)		Experts, mean (95% CI)		Novices, mean (95% CI)	
	Overall	Video	Overall	Video	Overall	Video
Undifferentiated histology						
Accuracy (%)	92.7 (102/110)	100 (10/10)	71.6 (68.6–74.6)*	71.1	58.1 (53.4–62.8)**	48.8
Sensitivity (%)	87.3 (48/55)	100 (5/5)	85.8 (79.5–92.1)	88.5	60.8 (53.1–68.5)	43.1
Specificity (%)	98.2 (54/55)	100 (5/5)	65.9 (63.2–68.6)	65.8	57.4 (53.1–61.7)	48.0
PPV (%)	98.0 (48/49)	100 (5/5)	52.9 (46.8–59.2)	51.1	53.3 (42.5–64.1)	45.0
NPV (%)	88.5 (54/61)	100 (5/5)	90.3 (84.6–95.4)	91.1	62.9 (49.0–76.8)	52.5
Submucosal invasion						
Accuracy (%)	87.3 (96/110)	100 (10/10)	72.6 (70.6–74.6)*	77.8	63.9 (56.4–71.4)**	58.8
Sensitivity (%)	83.3 (45/54)	100 (4/4)	70.9 (65.9–75.9)	86.2	60.8 (53.1–68.5)	78.5
Specificity (%)	91.1 (51/56)	100 (6/6)	76.5 (73.4–79.6)	70.2	73.0 (61.6–84.4)	51.8
PPV (%)	90.0 (45/50)	100 (4/4)	77.6 (71.4–83.8)	80.6	77.2 (64.0–90.4)	75.0
NPV (%)	85.0 (51/60)	100 (6/6)	67.9 (58.8–0.77)	75.9	51.0 (38.6–63.5)	47.9
Lymphovascular invasion						
Accuracy (%)	76.4 (84/110)	100 (10/10)	69.7 (59.6–79.8)	72.2	64.8 (61.4–68.2)	62.5
Sensitivity (%)	30.0 (6/20)	100 (1/1)	33.7 (26.7–40.7)	33.3	28.9 (20.6–37.2)	62.5
Specificity (%)	86.7 (78/90)	100 (9/9)	86.7 (85.2–88.2)	76.5	87.5 (83.2–92.7)	62.5
PPV (%)	33.3 (6/18)	100 (1/1)	46.9 (31.5–62.3)	8.7	40.0 (16.9–63.1)	3.8
NPV (%)	84.8 (78/92)	100 (9/9)	68.4 (56.7–73.7)	91.8	68.1 (48.8–87.4)	96.0
Lymph node metastasis						
Accuracy (%)	87.7 (71/81)	62.5 (5/8)	72.3 (65.8–78.8)*	68.1	67.7 (49.8–85.6)	65.6
Sensitivity (%)	41.7 (5/12)	33.3 (1/3)	17.6 (11.5–23.7)	22.2	14.8 (5.2–24.4)	29.2
Specificity (%)	95.4 (66/69)	80.0 (4/5)	85.3 (72.3—98.4)	95.6	83.4 (79.6–87.2)	87.5
PPV (%)	62.5 (5/8)	50 (1/2)	19.7 (3.9–35.5)	46.3	10.3 (2.6–18.0)	43.8
NPV (%)	90.4 (66/73)	66.7 (4/6)	81.8 (72.7–90.9)	67.9	76.2 (0.58–0.95)	70.5

*AI* artificial intelligence, *CI* confidence interval, *n* number of correct answers, *N* number of questions

^*^ P < 0.05, when accuracy was compared with that of the AI system using the Mcnemar’s test

^**^ *P* < 0.05, when the mean accuracy was compared with that of the experts using the Mann–Whitney U test

### External validation of the AI model

Table 4 shows the performance of the AI model in the external validation. The model demonstrated predictive accuracies (per patient) of 75.6% (sensitivity, 81.7%; specificity, 72.5%) for undifferentiated histology, 71.9% (sensitivity, 53.3%; specificity, 80.6%) for submucosal invasion, 88.8% (sensitivity, 31.8%; specificity, 94.5%) for LVI, and 87.0% (sensitivity, 10.0%; specificity, 98.5%) for LNM.

**Table 4:** External validation of trained AI model

Prediction target	Patient-based performance (n/N)
Undifferentiated histology	
Accuracy (%)	75.6 (183/242)
Sensitivity (%)	81.7 (67/82)
Specificity (%)	72.5 (116/160)
PPV (%)	60.4 (67/111)
NPV (%)	88.6 (116/131)
Submucosal invasion	
Accuracy (%)	71.9 (174/242)
Sensitivity (%)	53.3 (41/77)
Specificity (%)	80.6 (133/169)
PPV (%)	56.2 (41/73)
NPV (%)	78.2 (133/169)
Lymphovascular invasion	
Accuracy (%)	88.8 (215/242)
Sensitivity (%)	31.8 (7/22)
Specificity (%)	94.5 (208/220)
PPV (%)	36.8 (7/19)
NPV (%)	93.3 (208/223)
Lymph node metastasis	
Accuracy (%)	87.0 (131/153)
Sensitivity (%)	10.0 (2/20)
Specificity (%)	98.5 (129/133)
PPV (%)	50.0 (2/4)
NPV (%)	86.6 (129/149)

*AI* artificial intelligence, *PPV* positive prediction value, *NPV* negative prediction value, *n* number of correct predictions, *N* number of patients

Representative videos of the AI model in the test set and external validation are shown in Video 1 and 2, respectively. The resolution of images and videos in the external dataset (640 × 480) was lower than that in the internal dataset (1920 × 1080), owing to differences in the picture archiving and communication system between the two hospitals.

## Discussion

In this study, we developed and evaluated an AI model that predicts postoperative pathologic results of EGC based on conventional white-light endoscopic images and videos. The performance of the model was compared with that of endoscopists in a test and externally validated using videos from another institution.

Categorizing the differentiation status of EGC is pivotal in deciding the indication for ESD, considering the significantly lower curative resection rate in undifferentiated-type EGC compared to differentiated-type EGC [22, 23]. Since approximately 18% of undifferentiated-type EGC can initially be misclassified as differentiated-type with forceps biopsy, endoscopic features of the lesions, including ME-NBI, must be combined for accurate diagnosis [24–26]. In a previous study, an AI model trained with ME-NBI showed an accuracy of 86.2% for classifying EGC differentiation status [27]. In our study, the AI model exhibited an accuracy of 89.7% in white-light endoscopic videos and outperformed the experts in identifying undifferentiated-type EGC. These results suggest that AI can assist endoscopists in predicting the differentiation status, with both white-light and ME-NBI endoscopic images.

Although EUS is commonly used to detect submucosal invasion in EGC, its advantages over conventional endoscopy are insignificant, with an accuracy of approximately 70% [10]. Notably, these findings are consistent with our results, where the experts showed a mean accuracy of 72.6% for predicting submucosal invasion of EGC in the test. In contrast, the AI model demonstrated significantly higher accuracy than the experts. Therefore, endoscopic findings indicative of submucosal invasion in EGC, such as clubbing, abrupt cutting or fusion of folds, uneven or nodular depression, and remarked redness of surface can be assessed without ultrasound [28–31], and AI enhances this process by learning an extensive dataset of conventional endoscopic images.

Several studies have investigated deep learning-based prediction of submucosal invasion in EGC using endoscopic images, reporting accuracies ranging from 84 to 94% [16, 32–34]. However, two studies revealed that undifferentiated-type EGC was associated with lower predictive accuracies compared with differentiated-type EGC [19, 35], a tendency also observed in our study. Furthermore, the significantly lower sensitivity for submucosal invasion was observed in undifferentiated EGC. Given that submucosal invasion with undifferentiated histology indicates non-curative ESD in EGC, these findings suggest that endoscopists still need to be more conservative when deciding to perform ESD for undifferentiated-type EGC than for differentiated-type EGC, even with the assistance of AI.

The lack of research on predicting LNM from endoscopic images using AI can be attributed to the low incidence of LNM in patients with EGC. The LNM rates have been reported to be < 9% for mucosal cancer and < 20% for submucosal cancer, according to large-scale studies based on surgical specimens of EGC [36, 37]. In addition, the LVI rate of EGC was approximately 13% in another study based on surgical specimens [38]. Although our study included as many surgical patients as possible, these inherently low rates of LVI and LNM in EGC induced an imbalance between positive and negative cases within the datasets. This is the reason our model exhibited lower sensitivity and positive prediction value, resulting in a low AUC value compared to its high accuracy in predicting LVI and LNM. However, excluding some patients with negative LVI or LNM to address this data imbalance could introduce significant selection bias. Therefore, despite the potential effect of data imbalance, we chose to include patients consecutively in the study.

Additionally, the absence of a significant difference in the mean accuracy between experts and novices suggests that the ability to detect LNM does not necessarily improve with clinical experience. However, the AI model in our study showed higher accuracy and sensitivity than the experts in predicting LNM. One possible explanation for this is that the AI may have adapted to associate a deeper invasion depth with an increased probability of LNM in EGC. This is supported by the fact that the model showed increased sensitivity for LNM in submucosal cancer compared to mucosal cancer, with the highest sensitivity in cancer with SM2 invasion. This trend was also observed in the prediction of LVI, a pathologic factor correlated with LNM in EGC [18, 39]. However, within the same categories of mucosal or submucosal cancer, there appeared to be no specific endoscopic features suggestive of LNM. Moreover, there are factors other than endoscopic findings associated with LNM in EGC, including age, sex, and tumor size, as reported in previous studies [40–42]. Therefore, future research could focus on integrating various clinical features indicative of LNM with endoscopic images to enhance AI-based detection of LNM in EGC.

This study has several limitations. First, the AI model was trained using retrospective images after the selection process, potentially introducing bias into our study. To compensate for this, we also included videos from patients enrolled prospectively under the same indications for ESD and found consistent performance of the model between images and videos. Furthermore, we tested the performance of the model by comparing it with experts from various hospitals across the nation. Second, the predictive performance of the AI model was lower in the external validation than in the internal tests. This discrepancy can be partially explained by the inferior resolution of images and videos in the external dataset compared to those in the internal dataset. Additionally, previous studies reported the “overfitting effect” in AI, where the learning process becomes excessively adapted to the training data [43, 44]. Several studies on deep learning-based prediction of invasion depth in gastric neoplasms have also reported significant differences in accuracies between internal and external tests [19, 45, 46]. Nevertheless, the external validation of our model showed predictive accuracy above 70% for invasion depth, which was higher than reported predictive accuracy of EUS. The performance could be further improved by training the model with images from various institutions in the future. Third, this study did not evaluate ME-NBI images and videos of EGC. Training this model with NBI data can improve the histological diagnosis of EGC, and it is essential to train the model with NBI images and videos in further studies. Fourth, incorporating both ESD and surgical cases into the dataset may have affected the model’s performance due to heterogeneity among the data. The longer section intervals in surgical specimens compared to ESD specimens could potentially lead to underestimation of submucosal invasion and LVI in surgical specimens [47]. Finally, the findings of this study should be confirmed in randomized controlled trials, and we are planning to conduct prospective studies to apply this AI model in clinical practice.

In conclusion, this study suggests that AI has the potential to assist endoscopists in determining the optimal treatment strategy for EGC, showing high performance in predicting the differentiation status and invasion depth based on conventional endoscopic images and videos. However, the detection of LVI and LNM using deep learning-based methods requires further research.

## Supplementary Information

Below is the link to the electronic supplementary material.

Supplementary file1 (PNG 369 KB)

Supplementary file2 (PNG 3834 KB)

Supplementary file3 (DOCX 21 KB)

Supplementary file4 (DOCX 18 KB)

Supplementary file5 (DOCX 18 KB)

Supplementary file6 (MPG 157,906 KB)

Supplementary file7 (MPG 36,368 KB)
