# Correction: An artificial intelligence system for comprehensive pathologic outcome prediction in early gastric cancer through endoscopic image analysis (with video)

**DOI:** 10.1007/s10120-024-01549-8

**Published:** 2024-09-04

**Authors:** Seunghan Lee, Jiwoon Jeon, Jinbae Park, Young Hoon Chang, Cheol Min Shin, Mi Jin Oh, Su Hyun Kim, Seungkyung Kang, Su Hee Park, Sang Gyun Kim, Hyuk-Joon Lee, Han-Kwang Yang, Hey Seung Lee, Soo-Jeong Cho

**Affiliations:** 1 Department of Internal Medicine and Liver Research Institute, Seoul National University College of Medicine, 101 Daehak-Ro, Jongno-Gu, Seoul, 03080 Republic of Korea; 2 Ainex Corporation, Seoul, Republic of Korea; 3 Department of Internal Medicine, Seoul National University Bundang Hospital, Seoungnam-Si, Gyeonggi-Do, Republic of Korea; 4 Center for Health Promotion and Optimal Aging, Seoul National University Hospital, Seoul, Republic of Korea; 5 Department of Surgery, Seoul National University College of Medicine, Seoul, Republic of Korea; 6 Department of Pathology, Seoul National University College of Medicine, Seoul, Republic of Korea

**Correction: Gastric Cancer (2024) 27:1088–1099** 10.1007/s10120-024-01524-3

In this article, the affiliation details for Jiwoon Jeon, Jinbae Park were incorrectly given as “Ainex Co., LTD, Seoul, Republic of Korea” but should have been “Ainex Corporation, Seoul, Republic of Korea”.

The original article has been corrected.

