# Supplementary figures and images for: An artificial intelligence system for comprehensive pathologic outcome prediction in early gastric cancer through endoscopic image analysis (with video)
Source: Gastric Cancer. 2024 Jul 2;27(5):1088–99. doi: 10.1007/s10120-024-01524-3 (PMC11335909; doi:10.1007/s10120-024-01524-3)

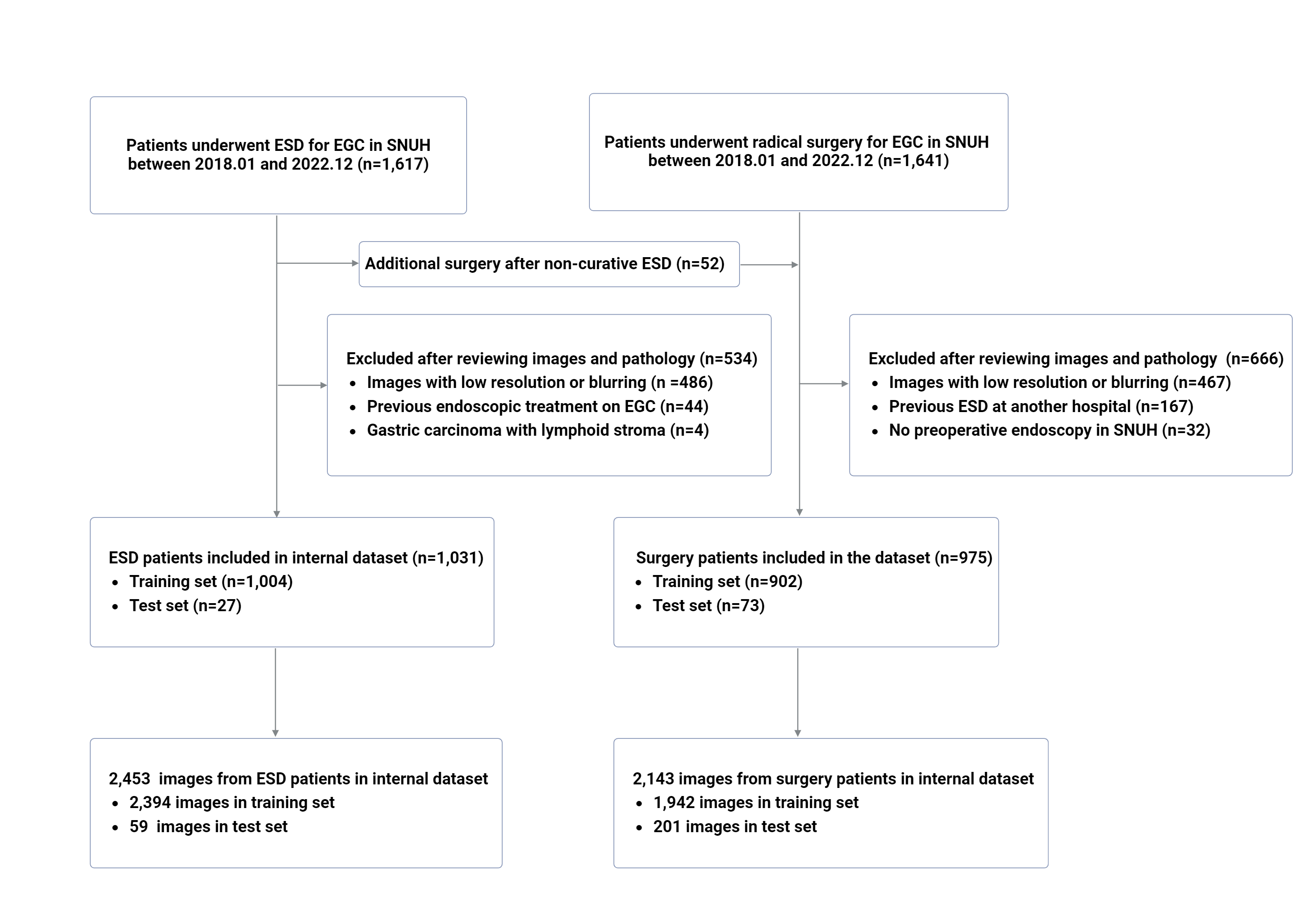

Supplement: Supplementary file 1 — Supplementary file1 (PNG 369 KB) [file 10120_2024_1524_MOESM1_ESM.png]

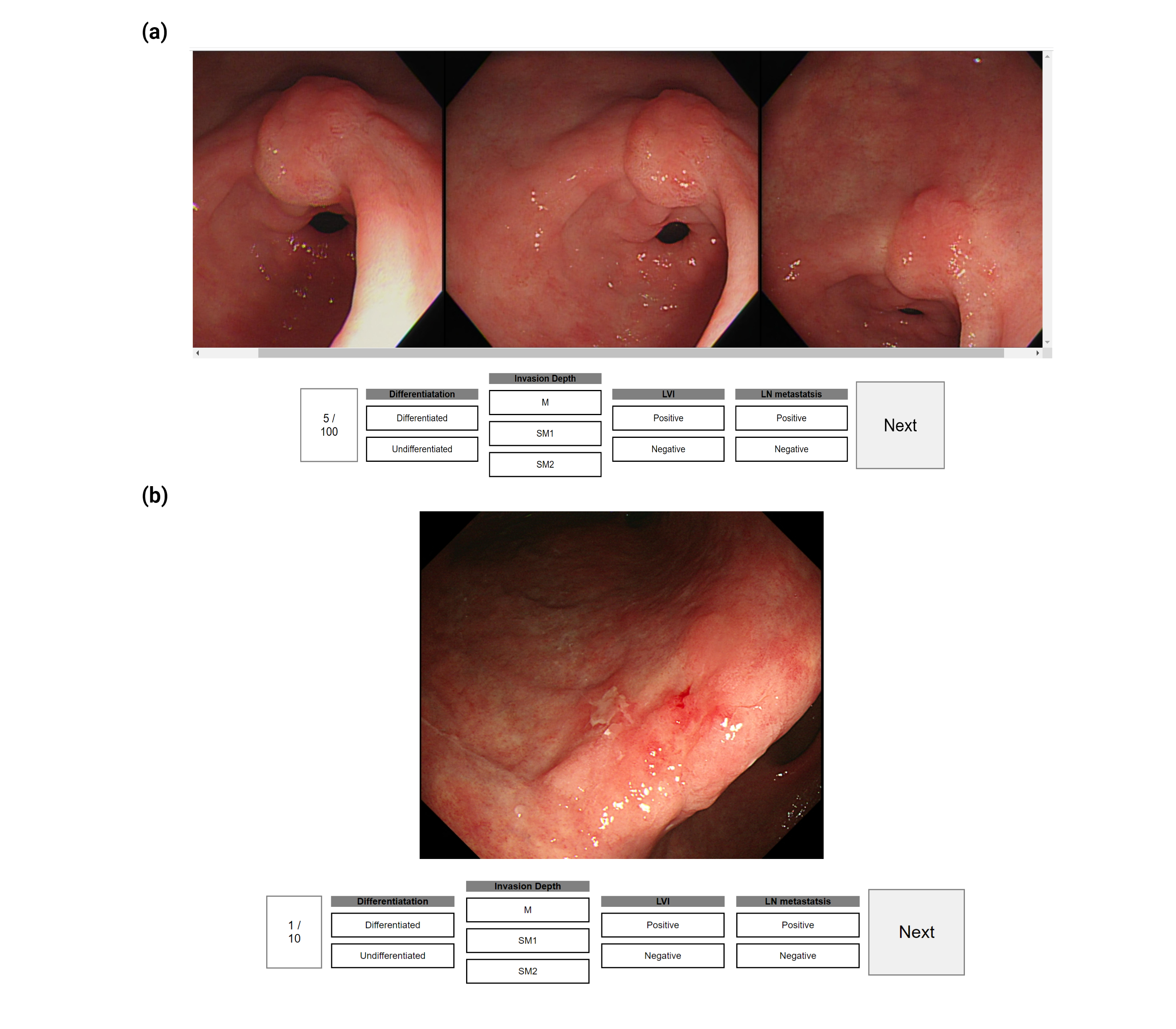

Supplement: Supplementary file 2 — Supplementary file2 (PNG 3834 KB) [file 10120_2024_1524_MOESM2_ESM.png]
