# Supplementary material for: An artificial intelligence system for comprehensive pathologic outcome prediction in early gastric cancer through endoscopic image analysis (with video)
Source: Gastric Cancer. 2024 Jul 2;27(5):1088–99. doi: 10.1007/s10120-024-01524-3 (PMC11335909; doi:10.1007/s10120-024-01524-3)
Supplement: Supplementary file 3 — Supplementary file3 (DOCX 21 KB) [file 10120_2024_1524_MOESM3_ESM.docx]

**Supplementary Table S1. Baseline clinicopathologic characteristics of patients in datasets**

| **Chacteristics** | **Internal dataset** | | **External dataset**  **(n=242)** |
| --- | --- | --- | --- |
|  | **Training set (n=2,059)** | **Test set**  **(n=110)** |  |
| Age, mean ± s.d. | 64.5 ± 10.7 | 63.6 ± 10.9 | 63.5 ± 10.8 |
| Male sex, n (%) | 1,414 (68.7) | 65 (59.1) | 167 (69.0) |
| Tumor size (mm), mean ± s.d. | 21.0 ± 13.8 | 24.3 ± 12.3 | 22.7 ± 14.5 |
| Data type, n (%) |  |  |  |
| Image | 1,906 (92.6) | 100 (90.9) | 153 (63.2) |
| Video | 153 (7.4) | 10 (9.1) | 89 (36.8) |
| Operation type, n (%) |  |  |  |
| ESD | 1,051 (51.0) | 29 (26.4) | 89 (36.8) |
| Surgery | 1,008 (49.0) | 81 (73.6) | 153 (63.2) |
| Location, n (%) |  |  |  |
| Cardia / Fundus / High body | 251 (12.2) | 14 (12.7) | 34 (14.0) |
| Low body / Mid body / Angle | 849 (31.2) | 54 (49.1) | 64 (26.4) |
| Antrum / Pylorus | 959 (46.6) | 42 (38.2) | 144 (59.5) |
| Gross type, n (%) |  |  |  |
| Elevated (type 0-I / 0-IIa) | 307 (14.9) | 23 (20.9) | 53 (22.0) |
| Flat (type 0-IIb) | 710 (34.5) | 40 (38.2) | 26 (10.8) |
| Depressed (type 0-IIc / 0-III) | 1,042 (50.6) | 45 (40.9) | 162 (67.2) |
| Invasion depth, n (%) |  |  |  |
| Mucosa | 1,527 (74.3) | 56 (50.9) | 164 (68.0) |
| Submucosa |  |  |  |
| SM1 | 151 (7.3) | 16 (14.5) | 30 (12.4) |
| SM2 | 378 (18.4) | 38 (34.5) | 47 (19.5) |

Abbreviations: ESD, endoscopic submucosal dissection; SM1, submucosal invasion being <500 µm; SM2, submucosal invasion being ≥500 µm
