# Supplementary material for: An artificial intelligence system for comprehensive pathologic outcome prediction in early gastric cancer through endoscopic image analysis (with video)
Source: Gastric Cancer. 2024 Jul 2;27(5):1088–99. doi: 10.1007/s10120-024-01524-3 (PMC11335909; doi:10.1007/s10120-024-01524-3)
Supplement: Supplementary file 4 — Supplementary file4 (DOCX 18 KB) [file 10120_2024_1524_MOESM4_ESM.docx]

**Supplementary Table S2. Performance of the AI model according to differentiation status of early gastric cancer.**

|  | Differentiated-type, mean (95% CI) | Undifferentiated-type, mean (95% CI) | *P* value |
| --- | --- | --- | --- |
| Submucosal invasion |  |  |  |
| Accuracy (%) | 91.1 (90.4 – 91.8) | 87.5 (86.4 – 88.6) | 0.008 |
| Sensitivity (%) | 82.3 (79.5 – 85.1) | 79.3 (76.3 – 82.2) | 0.016 |
| Specificity (%) | 89.2 (86.9 – 91.5) | 93.3 (89.7 – 96.9) | 0.222 |
| PPV (%) | 71.6 (64.3 – 78.9) | 73.6 (64.3 – 82.9) | 0.530 |
| NPV (%) | 93.8 (92.3 – 95.3) | 95.5 (92.7 – 98.3) | 0.222 |
| Lymphovascular invasion |  |  |  |
| Accuracy (%) | 83.5 (78.2 – 88.8) | 88.1 (82.6 – 93.6) | 0.095 |
| Sensitivity (%) | 27.3 (17.4 – 37.2) | 10.4 (3.7 – 17.1) | 0.008 |
| Specificity (%) | 95.4 (94.2 – 96.6) | 98.6 (96.9 – 100) | 0.008 |
| PPV (%) | 53.7 (43.0 – 64.4) | 53.7 (37.6 – 69.9) | 0.996 |
| NPV (%) | 86.2 (80.6 – 91.8) | 89.0 (83.8 – 94.2) | 0.421 |
| Lymph node metastasis |  |  |  |
| Accuracy (%) | 90.1 (86.0 – 94.2) | 83.8 (81.7 – 85.9) | 0.016 |
| Sensitivity (%) | 29.7 (14.8 – 44.6) | 26.9 (10.6 – 42.8) | 0.841 |
| Specificity (%) | 96.3 (92.6 – 100) | 92.6 (87.3 – 97.9) | 0.222 |
| PPV (%) | 50.2 (35.3 – 65.1) | 34.4 (14.5– 54.3) | 0.222 |
| NPV (%) | 94.0 (90.5 – 97.5) | 89.5 (84.0 – 95.0) | 0.095 |

Abbreviations: AI, artificial intelligence; PPV, positive prediction value; NPV, negative prediction value; CI, confidence interval
