# Supplementary material for: An artificial intelligence system for comprehensive pathologic outcome prediction in early gastric cancer through endoscopic image analysis (with video)
Source: Gastric Cancer. 2024 Jul 2;27(5):1088–99. doi: 10.1007/s10120-024-01524-3 (PMC11335909; doi:10.1007/s10120-024-01524-3)
Supplement: Supplementary file 5 — Supplementary file5 (DOCX 18 KB) [file 10120_2024_1524_MOESM5_ESM.docx]

**Supplementary Table S3. Performance of the AI model according to invasion depth of early gastric cancer.**

|  | Mucosal,  mean (95% CI) | Submucosal,  mean (95% CI) | SM2 invasion,  mean (95% CI) |
| --- | --- | --- | --- |
| Lymphovascular invasion |  |  |  |
| Accuracy (%) | 89.6 (87.2 – 92.0) | 63.0 (54.9 – 71.1) | 64.7 (53.5 – 75.9) |
| Sensitivity (%) | 3.0 (0.0 – 7.1) | 35.9 (26.9 – 44.9) | 41.4 (29.5 – 53.3) |
| Specificity (%) | 98.6 (97.9 – 99.3) | 83.3 (78.0 – 88.6) | 81.5 (70.9 – 92.1) |
| PPV (%) | 20.5 (5.6 – 35.4) | 61.7 (51.9 – 71.5) | 63.7 (51.5 – 75.8) |
| NPV (%) | 90.7 (87.8 – 93.6) | 63.4 (55.3 – 71.5) | 65.2 (53.5 – 76.9) |
| Lymph node metastasis |  |  |  |
| Accuracy (%) | 96.1 (94.4 -97.8) | 72.5 (68.9 – 76.1) | 73.3 (68.3 – 78.3) |
| Sensitivity (%) | 9.4 (0.0 – 25.4) | 38.7 (23.8 – 53.6) | 39.9 (25.0 – 54.8) |
| Specificity (%) | 97.4 (94.9 – 99.9) | 81.4 (72.1 – 90.7) | 82.4 (72.1 – 90.7) |
| PPV (%) | 7.0 (0.0 – 19.4) | 35.7 (20.8 – 50.6) | 32.2 (17.3 – 47.1) |
| NPV (%) | 98.7 (97.7 – 99.7) | 83.9 (72.7 – 95.1) | 85.7 (76.3 – 95.1) |

Abbreviations: AI, artificial intelligence; SM2, submucosal invasion being ≥500 µm; PPV, positive prediction value; NPV, negative prediction value; CI, confidence interval
